# Energy System Contributions in Repeated Sprint Tests: Protocol and Sex Comparison

**DOI:** 10.5114/jhk/175862

**Published:** 2024-02-17

**Authors:** Erkan Tortu, Tahir Hazir, Ayse Kin-Isler

**Affiliations:** 1 Faculty of Sport Sciences, Department of Coaching Education, Trabzon University, Trabzon, Turkiye.; 2 Faculty of Sport Sciences, Department of Exercise and Sport Sciences, Division of Training and Movement Sciences, Hacettepe University, Ankara, Turkiye.

**Keywords:** alactic energy system, glycolysis, aerobic system, team sports

## Abstract

The aim of this study was to investigate the energy system contributions to different repeated sprint protocols and also to determine sex-related differences in these contributions. Sixteen men and fourteen women team sport athletes randomly performed two cycling repeated sprint protocols with the same total duration (10 x 6 s and 6 x 10 s). Relative peak power (RPP), relative mean power (RMP), performance decrement (PD), oxygen uptake (VO_2_), blood lactate (LA), heart rate (HR) and ratings of perceived exertion (RPEs) were measured. The contributions of energy systems were calculated from oxygen consumption and lactate values during rest, exercise and recovery phases based on mathematical methods. Findings indicate that men had higher RPP and RMP. RPP did not differ according to protocols, while RMP was higher in the 10 x 6 s protocol. The sex effect in PD was similar; however, it was higher in the 6 x 10 s protocol. The effects of protocols on the maximum HR and LA were similar; however, the 6 x 10 s protocol resulted in higher RPEs. In both protocols women had higher ATP-PCr and men had higher glycolytic system contribution with similar oxidative system contribution. In addition, the 10 x 6 s protocol had higher ATP-PCr system contribution and the 6 x 10 s protocol had higher glycolytic system and oxidative system contributions. In conclusion, the contribution of energy systems, physiological and performance variables showed variations according to sex and different protocols.

## Introduction

Repeated sprint ability (RSA) is an important fitness component especially in team sports in recent years. RSA is widely chosen as a training and a testing method because it is similar to the movement profiles of team sport athletes (Turner and Stewart, 2013). Previous research has primarily focused on the relative energy contribution from metabolic pathways during repeated sprint activities of various types (running, cycling or swimming) and duration ranging from 7 to 9 minutes (Baron et al., 2022; Bogdanis et al., 1996; Peyrebrune et al., 2014). Repeated sprint protocols differ in the number of sprints, as well as sprint and recovery duration during rest intervals (Ikutomo et al., 2018). The ratio between sprint and recovery time is critical in measuring performance in repeated sprint tests and generally, short recovery (≤30 s) is applied between sprints (Gharbi et al., 2015).

Findings about energy metabolism during repeated sprints, which include various components in terms of physiological systems, are primarily limited to a small number of studies utilizing the muscle biopsy approach about 30 years ago (Bogdanis et al., 1996, 1998; Dawson et al., 1997). For instance, according to Gaitanos et al. (1993) Adenosine triphosphate-phosphocreatine (ATP-PCr) and glycolytic systems may contribute 55.9% and 44.1% of the energy demand, respectively, during a 6-s cycle exercise (Gaitanos et al., 1993). Girard et al. (2011) adjusted the previous results reporting an 8% contribution of the oxidative system during a 6-s maximal cycle exercise. Furthermore, Spencer et al. (2005) estimated the ATP-PCr and glycolytic contributions to be 65% and 32%, respectively (during a 3-s sprint). In contrast, Girard et al. (2011) estimated these rates to be 52% and 40% during a 3-s sprint and a 6-s sprint, respectively. The critical flaw in the approach utilized in that study was the estimation of PCr contribution during rest intervals. The time integral of oxygen consumption (VO_2_) was used to analyze PCr contribution because a period of 30 s was insufficient to identify the fast phase of excess post-exercise oxygen consumption (EPOC). Post-sprint VO_2_ may, however, represent both the resynthesis of PCr and the replenishment of oxygen stores. While the replenishment of oxygen stores during the fast phase of EPOC may account for up to 500 mL of oxygen (about 10 kJ) (Davis et al., 2014), the amount replenished in 30 s is uncertain. Given that the resynthesis of PCr stores is likely to take up the majority of the 30-s rest intervals between sprints (Bogdanis et al., 1995; Dawson et al., 1997; Gaitanos et al., 1993) utilizing the post-sprint VO_2_-time integral to estimate the PCr contribution appears reasonable. Furthermore, this is the most used non-invasive method available for determining the contributions of three different energy systems, and it has been used in some unique studies (Franchini et al., 2016; Ulupınar et al., 2023). Short recovery intervals, increased repetitions or increased sprint distance may result in increased oxidative system contribution during sprints, lowering performance due to reduced energy generation per unit time (Girard et al., 2011). During rest intervals in repeated-sprint exercises, the oxidative system is predicted to contribute mainly to the resynthesis of PCr stores (Dawson et al., 1997; Lopes-Silva et al., 2019). As a result, the oxidative system contributes minimally during sprints and maximally during rest intervals.

In recent years, there has been increasing interest in sex related changes in physiological and performance characteristics. It is well documented that men have greater muscle strength and higher power output compared to women (Billaut and Smith, 2009; Soydan et al., 2018). However, research has also indicated that women have greater resistance to fatigue than men (Bishop, 2012). The proposed mechanisms for sex differences in performance and fatigue resistance might be related to differences in muscle and fat mass, muscle metabolism that might include hormonal regulation and substrate utilization, the discharge rate of motor units and muscle morphology (Bishop, 2012). For instance, a large proportion of type II fibers are activated during maximal sprints (Toti et al., 2013) and women have lower power output than men due to a smaller cross-sectional area (Miller et al., 1993). Moreover, exercise-related energy metabolism differs between men and women and women were found to have a lower dependency on glycogen than men while exercising at the same intensity (Oosthuyse and Bosch, 2012). In repeated sprints, men have higher power output than women (Soydan et al., 2018) and the fact that the contribution from anaerobic-based energy systems is higher in men during repeated sprints may be another reason explaining this sex difference (Fomin et al., 2012). In addition, men have higher lactate levels during repeated sprint tests and this can be explained by their higher type II muscle fiber activation levels and higher glycolytic levels (Miller et al., 1993). Another explanation for women having lower lactate levels in repeated sprints than men might be related to a lower activity level of lactate dehydrogenase and phosphofructokinase (Jeon et al., 2019).

In team sports, performance is determined by physiological and technical skills, energy output, and efficiency. Therefore, understanding the metabolic responses in these circumstances is essential to improve targeted energy systems in training programs involving repeated-sprint protocols for both sex. Previous studies have mainly focused on performance variables, fatigue, recovery processes and sex differences during repeated sprints (Archiza et al., 2020; Bogdanis et al., 1996, 1998; Soydan et al., 2018). However, there is limited information about the contributions of energy systems during different protocols and sex differences in these contributions. Hence, this study was designed to investigate the energy system contribution to different repeated sprint protocols and also to determine sex differences in this contribution. We hypothesized that variations in the work-to-rest ratio and repeated sprint designs might result in differences in the energy system contributions and these differences might vary according to sex.

## Methods

### Participants

Thirty volunteer team sport athletes (14 women, 16 men) with no current musculoskeletal disorders and with at least three years of experience in team sports in various national leagues participated in this study. They were in the competitive season of the official national leagues (basketball: 5 men and 4 women, volleyball: 6 men and 5 women, handball: 5 men and 5 women). Participants were informed about the study's aims, procedures and possible risks, and signed written informed consent. They were also familiarized with the equipment and protocols before the testing sessions. This study was approved by the Hacettepe University Non-Interventional Clinical Researches Ethics Board (protocol code: 2019/20; date of approval: 03 September 2019) and this research abided by the guidelines of the Declaration of Helsinki.

### Design

#### Experimental Protocol

Participants were tested on three separate sessions with at least 48-h intervals at the same time of the day (10.00–11.00 am ± 1 h). The first session included a graded-exercise test to exhaustion for determination of VO_2max_. In the subsequent two sessions, participants completed either 6 x 10 s or 10 x 6 s cycle-RSA protocols with 30-s rest intervals in randomized order.

For women participants, menstruation was not considered since it was found that repeated sprint performance was not affected by the menstrual cycle (Wiecek et al., 2016). Relative peak power (RPP), relative mean power (RMP), performance decrement (PD), VO_2_, heart rate (HR), blood lactate concentration (BLa) and ratings of perceived exertion (RPEs) were measured during the RSA tests. Baseline VO_2_ and BLa were measured before the warm-up in a stationary sitting position.

### Procedures

#### Anthropometric Measurements

Body mass, body fat (%), fat mass (FM), and fat-free mass (FFM) were measured using a multifrequency bioelectrical impedance analyzer (Tanita MC-780, Japan), with 0.1-kg accuracy, and body height was measured to the nearest 0.1 cm using a portable stadiometer (Holtain, London, UK).

#### VO_2max_ Test

VO_2max_ was measured with a mobile cardiopulmonary exercise test system (Cosmed K_5_, Italy), which is capable of automatic gas analysis from each expiratory breath, with a ramp protocol on a cycle ergometer. Prior to each test, a sample of known gases (5.0% CO_2_ and 16.0% O_2_) was used to calibrate the portable metabolic gas analyzer. Tests were performed under standardized conditions (18–23°C and relative humidity below 70%). A four-minute warm-up was performed with the cycling power set at 50 W and speed at 60 rpm. Afterwards, the test was started at 50 W and 80 rpm, and cycling power was increased by 25 W every minute until 200 W was reached. After reaching 200 W, power increases of 25 W occurred every 2 minutes, and the test was terminated when the speed remained below 80 rpm for more than 10 s.

#### Cycling Repeated Sprint Tests

6 x 10 s and 10 x 6 s repeated sprint tests with 30-s rest intervals (Girard et al., 2011), which included the same total test duration and a different work-rest ratio, were performed on a bicycle ergometer (894E, Monark, Vansbro, Sweden) with the load of 10% of body weight. The inertial momentum of the pedal was not taken into consideration in calculating the measured power output since it is recommended to use higher resistive loads without acceleration if there is no chance of correcting inertia (Bogdanis et al., 2008). All tests were performed after a standardized warm-up (5 x 30 s at 100 W) and were initiated with the dominant leg after 5 min of rest. Performance variables for the RSA tests were as follows:

Peak power output (PP): the highest power output in each sprint cycle.

Mean power output (MP): the average power output reached in each sprint cycle.

Performance decrement (PD): the percentage of decrement in the power output was calculated with the following formula (Oliver, 2009):

PD = 100 x (1-total peak power/ideal peak power)

Ideal peak power: Peak power output x repetition number.

#### Physiological Measurements

VO_2_ levels were monitored at rest (10 min), during exercise, and recovery (15 min) phases in a breath-by-breath mode using a COSMED K_5_ (Rome, Italy) portable gas-exchange system. Before tests, the gas analyzer was calibrated by a sample of known gases (5.0% CO_2_ and 16.0% O_2_). A hand-held portable analyzer (Lactate Plus, Nova Biomedical, USA) was used to measure BLa values before (at rest) and 1, 3, 5, 7, and 10 min after the RSA tests. The HR was measured with HR monitors (Garmin, ABD) at 1-s intervals. The peak HR (HR_peak_) was determined as the highest HR that the participant reached during each RSA test. The RPE was determined using a Borg scale which was shown to participants immediately after the last sprint of each RSA test.

#### Determination of Energy Demand and Energy Systems Contribution

Oxidative, glycolytic, and ATP-PCr system contributions were estimated through body weight, VO_2_, BLa and the fast component of EPOC. The oxidative system was calculated as VO_2_ during exercise minus baseline VO_2_ by adjusting the exercise duration (La Monica et al., 2020). To estimate the contribution of the oxidative system, the total VO_2_ during the exercise phase was determined by calculating the area under the curve using a trapezoidal method. During the estimation of the glycolytic system, it was assumed that the accumulation of 1 mmol·L^−1^ of BLa was equivalent to 3 mL of O_2_ per kg of body weight (di Prampero and Ferretti, 1999). The contribution of the ATP-PCr system was calculated using the fast component of EPOC following the last sprint and the sum of the VO_2_-time integral during the rest intervals between the sprints (Latzel, 2018), since it is known that rest intervals between consecutive sprints are mainly devoted to replenish PCr stores (Dawson et al., 1997; Gaitanos et al., 1993). Total energy expenditure (TEE) was calculated as the sum of energy derived from oxidative, glycolytic, and ATP-PCr systems (Franchini et al., 2011). Relative energy expenditure (REE) was calculated by dividing the total energy expenditure by the duration of the protocols and oxygen demand (L) obtained from three energy systems were converted into energy equivalents assuming 20.92 kJ for each 1 liter of O_2_ (Gastin, 2001). The kinetics of EPOC was determined by a mono-exponential model using OriginPro 2019 software (OriginLab Corp., Northampton, USA). Thus, PCr contribution during the fast component of EPOC kinetics was calculated by multiplying the amplitude of the mono-exponential model by the time constant (Franchini et al., 2011).

### Statistical Analysis

After completing descriptive statistics of all variables, the Shapiro-Wilk test was used for verification of data normality. Differences in body composition and VO_2max_ test results of men and women participants were calculated using the independent samples *t*-test. Cohen’s *d* was used as effect sizes for the independent samples *t*-test and was classified according to Hopkins et al. (2009). The 2 x 2 (sex x protocol) mixed analysis of variance (ANOVA) with repeated measures was used to compare the variables related to different RSA protocols and sex. In addition, 2 x 3 (sex x energy system) mixed ANOVA was used for determining differences among energy system contribution according to sex in each RSA protocol with Bonferroni post hoc analysis. Partial eta square values (η^2^) were calculated for effect size in ANOVA. ES values were considered n^2^_p_ ≤ 0.01 small, n^2^_p_ ≤ 0.06 medium, and n^2^_p_ ≤ 0.14 large (Lakens, 2013). The data were analyzed using the Statistical Package for the Social Sciences version 21.0 (IBM Corp., Armonk, NY, USA), and significance was set at *p* ≤ 0.05.

## Results

Descriptive characteristics of participants are given in Table 1. Significant differences were observed in all variables in favor of men, except age and experience. Men had higher absolute VO_2_ (L·min^−1^) (*p* = 0.000; *d* = 2.26), whereas no significant difference was observed for relative VO_2_ (ml·kg^−1^·min^−1^) (*p* = 0.657; *d* = 0.16).

**Table 1:** Descriptive characteristics of men and women athletes.

Variables	Men (n=16)	Women (n = 14)	*p*	*d*
Age (y)	22.06 ± 2.2	22.22 ± 2.72	0.860	0.06
Experience (y)	7.06 ± 1.61	7,00 ± 1.3	0.910	0.04
Height (cm)	182.6 ± 3.4	169.82 ± 2.3	0.000	4.4
Body weight (kg)	77.98 ± 11.57	60.16 ± 5.5	0.000	1.97
Body fat (%)	11.41 ± 5.02	20.19 ± 4.45	0.000	1.85
Fat Mass (kg)	9.27 ± 5.1	12.2 ± 3.15	0.070	0.69
Fat Free Mass (kg)	68.71 ± 8.18	47.96 ± 4.49	0.000	3.14
Body Mass Index (kg/m^2^)	23.38 ± 2.34	20.86 ± 1.96	0.000	1.16
VO_2max_ (L·min^−1^)	3.24 ± 0.41	2.46 ± 0.42	0.000	2.26
VO_2max_ (ml•kg^−1^•min^−1^)	41.9 ± 4.93	41.2 ± 4.1	0.657	0.16

Note. Values are means ± standard deviations

## Mechanical and Physiological Variables

As can be seen in Table 2, significant sex effects were observed in RPP (F_(1;28)_ = 67.192; *p* = 0.000; *η^2^* = 0.706), while the protocol effect and sex x protocol interaction were not significant (*p* > 0.05). In terms of the significant sex effect, men had higher RPP values than women. In terms of RMP, significant sex (F_(1;28)_ = 20.622; *p* = 0.000; *η^2^* = 0.424) and protocol effects (F_(1;28)_ = 265.686; *p* = 0.000; *η^2^* = 0.905) were observed together with significant sex x protocol interaction (F_(1;28)_ = 9.443, *p* = 0.005, *η^2^* = 0.252). Men had higher RMP than women. Both women and men achieved higher power values during the 10 x 6 s protocol.

**Table 2:** Performance variables and physiological responses during the repeated-sprint protocols.

	10 × 6 s		6 × 10 s		Sex effect	Protocol effect	Sex × Protocol Interaction
	Men	Women	Men	Women			
RPP (W·kg^−1^)	14.84 ± 1.14	11.29 ± 1.4	14.86 ± 1.39	11.22 ± 1.22	F = 67.192*	F = 0.017	F = 0.056
RMP (W·kg^−1^)	10.61 ± 0.83	8.8 ± 1.16	9.31 ±. 68	7.92 ± 1.20	F = 20.622*	F = 265.686*	F = 9.443*
PD (%)	30.84 ±5 .88	27.7 ± 5.37	41.74 ± 8.59	40.7 ± 7.09	F = 0.872	F = 104.703*	F = 0.806
La_max_ (mmol·L^−1^)	12.18 ± 2.83	10.02 ± 1.94	13.16 ± 2.33	12.05 ± 2.95	F = 3.768	F = 14.558*	F = 1.763
HRpeak (bpm)	162.54 ± 15.82	163.06 ± 18.67	166.01 ± 10.39	164.91 ± 9.14	F = 0.005	F = 0.683	F = 0.620
RPE	13.38 ± 1.2	15.14 ± 095	16 ± 1.1	17.64 ± 0.5	F = 26.405*	F = 318.334*	F = 0.1888

RPP=Relative Peak Power, RMP = Relative Mean Power, PD = Performance Decrement, La_max_ = Maximal lactate, HR = Heart rate, RPE = Ratings of perceived exertion. ^*^ *p* < 0.05 *Note*. Values are means ± standard deviations

For PD, there was a significant protocol effect (F_(1;28)_ = 104.703; *p* = 0.000; *η^2^* = 0.789) which was due to higher PD during the 6 x 10 s protocol. Sex effect and sex x protocol interaction were not significant (*p* > 0.05) indicating that men and women were not different in terms of PD during different RSA protocols.

Considering physiological responses, a significant protocol effect (F_(1;28)_ = 14.558; *p* = 0.001; *η^2^* = 0.342) was observed for maximum blood lactate (La_max_), while sex effect and sex x protocol interaction were not significant (*p* > 0.05). Both men and women had higher La_max_ responses after the 6 x 10 s protocol. For HR_peak_, no significant sex effect, protocol effect and sex x protocol interaction were observed (*p* > 0.05). In addition, for the RPE, the sex effect (F_(1;28)_ = 26.405; *p* = 0.000; *η^2^* = 0.485) and the protocol effect (F_(1;28)_ = 318.334; *p* = 0.000; *η^2^* = 0.919) were significant with no significant sex x protocol interaction (*p* > 0.05). Women had higher RPE responses than men.

### Energy System Contributions

Regarding energy expenditure and estimated absolute energy contributions (Table 3), the sex effect was significant for ATP-PCr (F_(1;28)_ = 14.482, *p* = 0.001, *η^2^* = 0.341) and glycolytic systems (F_(1;28)_ = 35.320, *p* = 0.000, *η^2^* = 0.558), whereas there was no significant effect on the oxidative system (*p* > 0.05). Men had higher ATP-PCr and glycolytic system contributions than women. In addition, protocol effects on ATP-PCr (F_(1;28)_ = 120.118, *p* = 0.000, *η^2^* = 0.811), glycolytic (F_(1;28)_ = 19.626, *p* = 0.000, *η^2^* = 0.412) and oxidative systems (F_(1;28)_ = 8.769, *p* = 0.006, *η^2^* = 0.238) were significant. In both men and women, the contribution of ATP-PCr system was higher in the 10 x 6 s protocol, whereas glycolytic and oxidative system contributions were higher in the 6 x 10 s protocol. Sex x protocol interaction was not significant in ATP-PCr, glycolytic and oxidative systems (*p* > 0.05).

**Table 3:** Metabolic energy variables during repeated-sprint protocols.

	10 × 6-s		6 × 10-s		Sex effect	Protocol effect	Sex × Protocol Interaction
	Men	Women	Men	Women			
ATP-PCr (kJ)	243.09 ± 54.57	192.55 ± 25.06	163.94 ± 30.3	128.94 ± 17.44	F = 14.482*	F = 120.118*	F = 1.423
Glycolytic (kJ)	54.23 ± 9.53	32.85 ± 7.72	59.27 ± 11.38	40.44 ± 10.95	F = 35.320*	F = 19.626*	F = 0.485
Oxidative (kJ)	15.83 ± 12.73	9.08 ± 7.46	18.35 ± 10.12	17.18 ± 6.38	F = 1.698	F = 8.769*	F = 2.422
TEE (kJ)	313.16 ± 64.91	237.47 ± 32.74	241.56 ± 69.02	186.55 ± 29.22	F = 19.802*	F = 67.878*	F = 2.664
REE (kJ^.^min^−1^)	59.94 ± 11.8	42.63 ± 5.95	69.02 ± 12.37	53.3 ± 8.35	F = 19.815*	F = 54.298*	F = 0.209

TEE = Total energy expenditure; REE = Relative energy expenditure ^*^p < 0.05

*Note*. Values are means ± standard deviations

For TEE and REE, sex effect (TEE: F_(1;28)_ = 19.802, *p* = 0.000, *η^2^* = 0.414; REE: F_(1;28)_ = 19.815, *p* = 0.000, *η^2^* = 0.414) and protocol effect (TEE: F_(1;28)_ = 67.878, *p* = 0.000, *η^2^ =* 0.708; REE: F_(1;28)_ = 54.298, *p* = 0.000, *η^2^* = 0.660) were significant with no significant sex x protocol interaction (*p* > 0.05). Men and women had higher TEE in the 10 x 6 s protocol, while REE was higher in the 6 x 10 s protocol. In addition, men had higher TEE and REE than women in both protocols.

**Figure 1:**
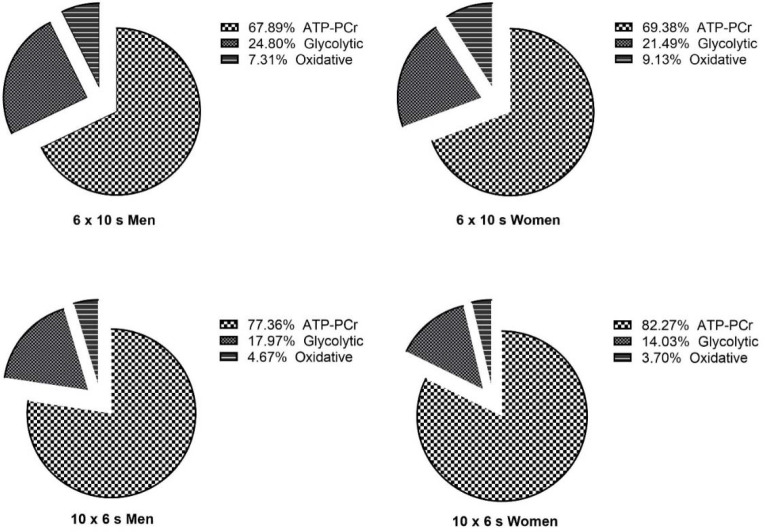
Estimated relative energy system contribution in 10 × 6 s and 6 × 10 s (sprints only) protocols.

For the estimated relative energy system contributions (Figure 1), the sex effect was significant on percentages of ATP-PCr (F_(1;28)_ = 152.789, *p* = 0.008, *η^2^* = 0.227) and glycolytic systems (F_(1;28)_ = 7.230, *p* = 0.012, *η^2^* = 0.205), whereas there was no significant effect on the percentage of the oxidative system (*p* > 0.05). While the percentage of ATP-PCr contribution was higher in women than men, the percentage of glycolytic system contribution was higher in men than in women. The effect of the protocol on percentages of ATP-PCr (F_(1;28)_ = 225.215, *p* = 0.000, *η^2^* = 0.889), glycolytic (F_(1;28)_ = 762.407, *p* = 0.000, *η^2^* = 0.801) and oxidative systems (F_(1;28)_ = 40.907, *p* = 0.000, *η^2^* = 0.594) contributions was significant. The 10 x 6 s protocol had higher contributions from the ATP-PCr and glycolytic systems, whereas the 6 x 10 s protocol had higher contributions from the oxidative systems in both men and women. Sex x protocol interaction was significant on percentages of ATP-PCr (F_(1;28)_ = 43.224, *p* = 0.030, *η^2^* = 0.157), and the oxidative system (F_(1;28)_ = 4.874, *p* = 0.036, *η^2^* = 0.148), whereas it was not significant for percentages of the glycolytic system contribution (*p* > 0.05). The significance of the interaction statistics for the percentage of ATP-PCr and oxidative systems indicated that the variation in the contributions of energy systems in the protocols was significantly different between the sexes.

## Discussion

This study is the first to compare energy system contribution as well as physiological and performance responses to different forms of RSA according to sex. Our hypothesis that variations in the work-to-rest ratio and repeated exercise designs would affect the amount of energy contributed by the energy systems overall or during rest intervals and would differ between sex was supported by the primary results.

As expected, men achieved higher relative and absolute power output in two cycle-based repeated sprint protocols, with no sex difference in PD, while the protocol effect on PD was significant. Moreover, the 6 x 10 s protocol resulted in higher PD than the 10 x 6 s protocol in both sexes. These results are similar to those of previous studies (Billaut et al., 2003; Soydan et al., 2018). For instance, in a study by Billaut and Bishop (2012) (20 x 5-s sprint with 25-s passive recovery), men achieved higher power output than women. Billaut et al. (2003) also reported that women had lower power output than men in repeated sprint tests performed with maximum effort on a cycle ergometer. It was therefore not surprising to see a considerable variation in power production between men and women (Billaut et al., 2003). Bogdanis et al. (1998) indicated that during 6- to 10-s cycling sprints, the glycolytic contribution to total ATP resynthesis was >40%, whereas the oxidative contribution was minimal. The lower glycolytic energy contribution in women compared to men during repeated sprints may result in lower anaerobic capacity in women and a more significant reduction in sprint performance in the final part of sprints. As indicated before, a large proportion of type II muscle fibers is activated during maximal cycle sprints (Toti et al., 2013) and a smaller cross-sectional area of type II fibers in women (Nuzzo, 2022) might be the reason why men achieved higher power values than women in both protocols. In addition, the non-significant difference in PD between men and women in both protocols is also in line with previous research. For instance, Soydan et al. (2018) also did not find any difference in PD after a 5 x 6-s cycle RSA test. It may be that the protocols used in the present study could not drive enough fatigue to reach significant sex difference in PD. In terms of significant protocol effect, higher PD in the 6 x 10 s protocol may be due to longer sprint time and a lower work-rest ratio in this protocol in which insufficient recovery time resulted in significant reductions in maintaining maximum performance.

The effect of protocols and sex on HR_max_ were similar and these results were in line with the findings of previous research. For instance, Soydan et al. (2018) found no significant differences in HR_max_ between men and women team sport athletes after 5 x 6 s cycling RSA tests. Similarly, Kappus et al. (2015) found no significant differences between men and women following a maximal exercise bout and Fomin et al. (2012) found no significant sex differences in HR_max_ in trained adolescents. These findings support our HR_max_ findings which indicated that men and women team sport athletes experienced similar physiological strain during the different RSA protocols. The 6 x 10 s protocol resulted in higher RPE values. This may be due to the increase in sprint time rather than the work-rest ratio and the number of repetitions, as in the lactate data. Previous research reported that the protocol effect on the RPE in men and women was significant because they could not get enough psychological rest during recovery (Little and Williams, 2007). It can be said that the 6 x 10 s protocol had an additional psychological effect of 120 s less resting time and resulted in higher RPE values.

Our results indicated similar La_max_ responses in men and women. Although men reached higher La_max_ levels in both protocols, this difference was not statistically significant. This result is dissimilar to results regarding the sex differences in previous studies which observed higher La_max_ levels in men (Soydan et al., 2018). On the other hand, men and women achieved higher La_max_ in the 6 x 10 s protocol compared to the 10 x 6 s protocol. The reason for reaching higher La_max_ in the 6 x 10 s protocol may be due to longer sprint time and a lower work-rest ratio. It was observed that sprints with a distance of more than 30 m or longer than 5 s induced higher blood lactate values (Little and Williams, 2007). For instance, Ulupınar et al. (2021) reported that in two different repeated sprint protocols with similar total sprint distances (10 x 40 m and 20 x 20 m), athletes showed higher lactate responses in the 10 x 40 m protocol compared to the 20 x 20 m protocol. Therefore, when other variables are kept constant, increasing sprint distance or duration or decreasing recovery time increases the lactate response to exercise (Ulupınar et al., 2021).

Cycling based RSA tests are frequently used to estimate the contribution of energy systems due to the convenience they provide in practice (Bogdanis et al., 1995; Dawson et al., 1997; Franchini et al., 2016; La Monica et al., 2020). In this study, the effects of sex and protocol on ATP-PCr and glycolytic system energy expenditure were significant. For the oxidative system contribution, the sex effect was similar, however, the protocol effect was significantly different. Given that the recovery of PCr stores is likely to be the primary focus of the 30-s rest intervals between sprints, the post-sprint VO_2_-time integral can be used to determine the contribution of PCr stores (Dawson et al., 1997). This method is presently the only non-invasive method for distinguishing between the contributions of three energy systems (Davis et al., 2014; Panissa et al., 2018; Ulupınar and Özbay, 2022). When the overall exercise duration is equalized, research indicates that VO_2_ during rest intervals may significantly alter energy system absolute and percentage contributions during intermittent sprint exercises (Gastin, 2001; Milioni et al., 2017). This research revealed significant differences in the impacts of the protocols on total sprint duration and the percentage of performance decrement (Table 2). In another study using a similar methodology, Ulupınar and Özbay (2022) reported that contributions of the ATP-PCr, glycolytic and oxidative energy systems were 68%, 17% and 14% following a 10 x 6 s sprint, respectively. In the present study, values of 77%, 18%, and 5% were found for men athletes and 82%, 14%, and 4% for women, respectively. It is possible that these differences might be due to different sample groups (indoor team sport vs. combat athletes) or the applied RSA test mode (Ulupınar and Özbay, 2022).

In the present study, during both repeated sprint protocols the highest energy contribution was provided by the ATP-PCr system in men and women. It is also known that work-rest periods in high-intensity and repeated exercise directly affect PCr resynthesis (Forbes et al., 2009). The study results support both a higher ATP-PCr system contribution and a lower percentage of decrement in performance during the 10 x 6 s protocol. The glycolytic system contribution was highest in the 6 x 10 s protocol in both sexes. In previous research, it was reported that the energy contribution from the glycolytic system was higher in RSA tests where the sprint time was more than 5 s (Gastin, 2001). The short recovery time causes a decrease in the energy contribution from the ATP-PCr system, and the energy requirement necessary to maintain performance is provided by the glycolytic system (Girard et al., 2011; Lopes-Silva et al., 2019; Turner and Stewart, 2013). Although the number of sprints is 40% less in the 6 x 10 s protocol, the 67% (4-s) increase in the sprint time and the 44% decrease in the work:rest ratio (1:3) might explain the increase in the contribution from the glycolytic system.

During repeated sprints, the main function of the oxidative energy system is to resynthesize PCr, remove accumulated inorganic phosphate, and oxidize lactate during short recovery times (Bogdanis et al., 1996; Dawson et al., 1997; Lopes-Silva et al., 2019). In order to maintain maximum performance, the oxidative energy system contribution must be low during sprints and high during recovery. In the present study, the increased sprint time of 67% in the 6 x 10 s protocol increased the contribution from the oxidative system; however, it caused a further decrease in performance. The reason why the oxidative contribution was lower in the present study might be due to differences in the training level or training history of athletes, as well as sprint time and the number of repetitions. In this study, the percentage of oxidative system contribution was 4.67–3.70% for men and women in the 10 x 6 s protocol and 7.31–9.13% in the 6 x 10 s protocol.

Similarly, previous studies reported that increasing sprint duration or sprint distance increases the energy contribution from the oxidative system, but causes a decrease in performance (Bishop, 2012; Girard et al., 2011). Girard et al. (2011) reported that the oxidative contribution was 8% during the first 6-s sprint and 40% during the 10^th^ sprint (Girard et al., 2011). Other studies investigating energy metabolism during 60-s maximal exercise reported that the oxidative contribution was around 45% (Gastin, 2001). Kishali et al. (2021) reported that the oxidative contribution in the 6-s single sprint, 60-s single maximal exercise, and 10 x 6-s repeated sprint exercise were 5%, 29%, and 14%, respectively.

TEE was lowest in the 6 x 10 s and highest in the 10 x 6 s protocol, and men had higher TEE than women. This sex difference was due to the lower O_2_ consumption of women in both protocols since it is known that energy metabolism associated with exercise varies between men and women. For instance, women often rely less on glycogen than men at similar exercise intensity (Oosthuyse and Bosch, 2012). In addition, previous studies reported that during 10 x 6-s repeated sprint tests performed by wrestlers and kickboxers, the total energy expenditure was 332 kJ and 409 kJ, respectively (Kishali et al., 2021; Ulupınar et al., 2021). Contrary to the studies mentioned above which used the same methodology, our study resulted in fewer energy demands. Team sports have lower anaerobic-based energy system contribution compared to wrestling and kickboxing which might be the reason for finding smaller energy demands.

## Conclusions

The present study demonstrated that the contribution of energy pathways was different between 10 x 6 s and 6 x 10 s RSA tests in both sexes. As a result, the highest contribution from anaerobic energy systems was seen in the sprint (10 x 6 s) protocol with more repetitions and shorter duration compared to the protocol with less repetitions and longer sprint duration (6 x 10 s). The effect of sex on the energy system contributions differed for the ATP-PCr and glycolytic energy systems, while there was no sex effect for the oxidative system contribution. In conclusion, these findings can be used in team sports to improve energy systems in target during training programs for both sexes.

## References

[ref1] Archiza, B., Andaku, D. K., Beltrame, T., Libardi, C. A., & Borghi-Silva, A. (2020). The Relationship Between Repeated-Sprint Ability, Aerobic Capacity, and Oxygen Uptake Recovery Kinetics in Female Soccer Athletes. Journal of Human Kinetics, 75, 115–126. 10.2478/hukin-2020-0042 33312300 PMC7706679

[ref2] Baron, J., Gupta, S., Bieniec, A., Klich, G., Gabrys, T., Szymon Swinarew, A., Svatora, K., & Stanula, A. (2022). The Effects of Two Different Rest Intervals on the Repeated Skating Ability of Ice Hockey Forwards and Defensemen. Journal of Human Kinetics, 84, 216–223. 10.2478/hukin-2022-0102 36457480 PMC9679177

[ref3] Billaut, F., & Bishop, D. J. (2012). Mechanical work accounts for sex differences in fatigue during repeated sprints. European Journal of Applied Physiology, 112(4), 1429–1436. 10.1007/s00421-011-2110-1 21830096

[ref4] Billaut, F., Giacomoni, M., & Falgairette, G. (2003). Maximal intermittent cycling exercise: effects of recovery duration and gender. Journal of Applied Physiology, 95(4), 1632–1637. 10.1152/japplphysiol.00983.2002 12794037

[ref5] Billaut, F., & Smith, K. (2009). Sex alters impact of repeated bouts of sprint exercise on neuromuscular activity in trained athletes. Applied Physiology, Nutrition, and Metabolism, 34(4), 689–699. 10.1139/H09-058 19767805

[ref6] Bishop, D. J. (2012). Fatigue during intermittent-sprint exercise. Clinical and Experimental Pharmacology and Physiology, 39(9), 836–841. 10.1111/j.1440-1681.2012.05735.x 22765227

[ref7] Bogdanis, G., Nevill, M., Lakomy, H., & Boobis, L. (1998). Power output and muscle metabolism during and following recovery from 10 and 20 s of maximal sprint exercise in humans. Acta Physiologica Scandinavica, 163(3), 261–272. 10.1046/j.1365-201x.1998.00378.x 9715738

[ref8] Bogdanis, G., Papaspyrou, A., Lakomy, H., & Nevill, M. (2008). Effects of inertia correction and resistive load on fatigue during repeated sprints on a friction-loaded cycle ergometer. Journal of Sports Sciences, 26(13), 1437–1445. 10.1080/02640410802209000 18923956

[ref9] Bogdanis, G. C., Nevill, M. E., Boobis, L. H., & Lakomy, H. (1996). Contribution of phosphocreatine and aerobic metabolism to energy supply during repeated sprint exercise. Journal of Applied Physiology, 80(3), 876–884. 10.1152/jappl.1996.80.3.876 8964751

[ref10] Bogdanis, G. C., Nevill, M. E., Boobis, L. H., Lakomy, H., & Nevill, A. M. (1995). Recovery of power output and muscle metabolites following 30 s of maximal sprint cycling in man. Journal of Physiology, 482(2), 467–480. 10.1113/jphysiol.1995.sp020533 7714837 PMC1157744

[ref11] Davis, P., Leithäuser, R. M., & Beneke, R. (2014). The energetics of semicontact 3× 2-min amateur boxing. International Journal of Sports Physiology and Performance, 9(2), 233–239. 10.1123/ijspp.2013-0006 24572964

[ref12] Dawson, B., Goodman, C., Lawrence, S., Preen, D., Polglaze, T., Fitzsimons, M., & Fournier, P. (1997). Muscle phosphocreatine repletion following single and repeated short sprint efforts. Scandinavian Journal of Medicine & Science in Sports, 7(4), 206–213. 10.1111/j.1600-0838.1997.tb00141.x 9241025

[ref13] di Prampero, P. E., & Ferretti, G. (1999). The energetics of anaerobic muscle metabolism: a reappraisal of older and recent concepts. Respiration Physiology, 118(2–3), 103–115. 10.1016/S0034-5687(99)00083-3 10647856

[ref14] Fomin, Å., Ahlstrand, M., Schill, H. G., Lund, L. H., Ståhlberg, M., Manouras, A., & Gabrielsen, A. (2012). Sex differences in response to maximal exercise stress test in trained adolescents. BMC Pediatrics, 12(1), 1–8. 10.1186/1471-2431-12-127 22906070 PMC3472286

[ref15] Forbes, S. C., Paganini, A. T., Slade, J. M., Towse, T. F., & Meyer, R. A. (2009). Phosphocreatine recovery kinetics following low-and high-intensity exercise in human triceps surae and rat posterior hindlimb muscles. American Journal of Physiology-Regulatory, Integrative and Comparative Physiology, 296(1), R161–R170. 10.1152/ajpregu.90704.2008 18945946 PMC2636983

[ref16] Franchini, E., Sterkowicz, S., Szmatlan-Gabrys, U., Gabrys, T., & Garnys, M. (2011). Energy system contributions to the special judo fitness test. International Journal of Sports Physiology and Performance, 6(3), 334–343. 10.1123/ijspp.6.3.334 21911859

[ref17] Franchini, E., Takito, M. Y., & Kiss, M. A. P. D. M. (2016). Performance and energy systems contributions during upper-body sprint interval exercise. Journal of Exercise Rehabilitation, 12(6), 535–541. 10.12965/jer.1632786.393 28119874 PMC5227314

[ref18] Gaitanos, G. C., Williams, C., Boobis, L. H., & Brooks, S. (1993). Human muscle metabolism during intermittent maximal exercise. Journal of Applied Physiology, 75(2), 712–719. 10.1152/jappl.1993.75.2.712 8226473

[ref19] Gastin, P. B. (2001). Energy system interaction and relative contribution during maximal exercise. Sports Medicine (Auckland, N.Z.), 31(10), 725–741. 10.2165/00007256-200131100-00003 11547894

[ref20] Gharbi, Z., Dardouri, W., Haj-Sassi, R., Chamari, K., & Souissi, N. (2015). Aerobic and anaerobic determinants of repeated sprint ability in team sports athletes. Biology of Sport, 32(3), 207–212. 10.5604/20831862.1150302 26424923 PMC4577558

[ref21] Girard, O., Mendez-Villanueva, A., & Bishop, D. (2011). Repeated-sprint ability-part I : factors contributing to fatigue. Sports Medicine (Auckland, N.Z.), 41(8), 673–694. 10.2165/11590550-000000000-00000 21780851

[ref22] Hopkins, W., Marshall, S., Batterham, A., & Hanin, J. (2009). Progressive statistics for studies in sports medicine and exercise science. Medicine Science in Sports Exercise, 41(1), 3–13. 10.1249/MSS.0b013e31818cb278 19092709

[ref23] Ikutomo, A., Kasai, N., & Goto, K. (2018). Impact of inserted long rest periods during repeated sprint exercise on performance adaptation. European Journal of Sport Science, 18(1), 47–53. 10.1080/17461391.2017.1383515 29032729

[ref24] Jeon, Y., Choi, J., Kim, H. J., Lee, H., Lim, J.-Y., & Choi, S.-J. (2019). Sex-and fiber-type-related contractile properties in human single muscle fiber. Journal of Exercise Rehabilitation, 15(4), 537–545. 10.12965/jer.1938336.168 31523674 PMC6732543

[ref25] Kishali, N., Ulupinar, S., & Ozbay, S. (2021). Energy system contributions and physiological responses during single and repeated Wingate exercise forms in kickboxers. Medicina dello Sport, 74(2), 223–234. 10.23736/S0025-7826.21.03808-4

[ref26] La Monica, M. B., Fukuda, D. H., Starling-Smith, T. M., Clark, N. W., & Panissa, V. L. (2020). Alterations in energy system contribution following upper body sprint interval training. European Journal of Applied Physiology, 120(3), 643–651. 10.1007/s00421-020-04304-w 31974857

[ref27] Lakens, D. (2013). Calculating and reporting effect sizes to facilitate cumulative science: a practical primer for t-tests and ANOVAs. Frontiers in Psychology, 4, 863. 10.3389/fpsyg.2013.00863 24324449 PMC3840331

[ref28] Latzel, R., Hoos, O., Stier, S., Kaufmann, S., Fresz, V., Reim, D., & Beneke, R. (2018). Energetic profile of the basketball exercise simulation test in junior elite players. International Journal of Sports Physiology and Performance, 13(6), 810–815. 10.1123/ijspp.2017-0174 29182413

[ref29] Little, T., & Williams, A. G. (2007). Effects of sprint duration and exercise: rest ratio on repeated sprint performance and physiological responses in professional soccer players. Journal of Strength & Conditioning Research, 21(2), 646–648. 10.1519/00124278-200705000-00063 17530972

[ref30] Lopes-Silva, J. P., da Silva Santos, J. F., Abbiss, C. R., & Franchini, E. (2019). Measurement properties and feasibility of repeated sprint ability test: A systematic review. Strength & Conditioning Journal, 41(6), 41–61. 10.1519/SSC.0000000000000495

[ref31] Milioni, F., Zagatto, A. M., Barbieri, R. A., Andrade, V. L., dos Santos, J. W., Gobatto, C. A., da Silva, A. S., Santiago, P. R. P., & Papoti, M. (2017). Energy systems contribution in the running-based anaerobic sprint test. International Journal of Sports Medicine, 38(03), 226–232. 10.1055/s-0042-117722 28192833

[ref32] Miller, A. E. J., MacDougall, J., Tarnopolsky, M., & Sale, D. (1993). Gender differences in strength and muscle fiber characteristics. European Journal of Applied Physiology and Occupational Physiology, 66(3), 254–262. 10.1007/BF00235103 8477683

[ref33] Nuzzo, J. L. (2022). Narrative Review of Sex Differences in Muscle Strength, Endurance, Activation, Size, Fiber Type, and Strength Training Participation Rates, Preferences, Motivations, Injuries, and Neuromuscular Adaptations. Journal of Strength & Conditioning Research, 37(2), 494–536. 10.1519/JSC.0000000000004329 36696264

[ref34] Oliver, J. L. (2009). Is a fatigue index a worthwhile measure of repeated sprint ability? Journal of Science and Medicine in Sport, 12(1), 20–23. 10.1016/j.jsams.2007.10.010 18078786

[ref35] Oosthuyse, T., & Bosch, A. N. (2012). Oestrogen's regulation of fat metabolism during exercise and gender specific effects. Current Opinion in Pharmacology, 12(3), 363–371. 10.1016/j.coph.2012.02.008 22398320

[ref36] Panissa, V. L., Fukuda, D. H., Caldeira, R. S., Gerosa-Neto, J., Lira, F. S., Zagatto, A. M., & Franchini, E. (2018). Is oxygen uptake measurement enough to estimate energy expenditure during high-intensity intermittent exercise? Quantification of anaerobic contribution by different methods. Frontiers in Physiology, 9, 868. 10.3389/fphys.2018.00868 30038583 PMC6046462

[ref37] Peyrebrune, M., Toubekis, A., Lakomy, H., & Nevill, M. (2014). Estimating the energy contribution during single and repeated sprint swimming. Scandinavian Journal of Medicine & Science in Sports, 24(2), 369–376. 10.1111/j.1600-0838.2012.01517.x 22897515

[ref38] Soydan, T. A., Hazir, T., Ozkan, A., & Kin-Isler, A. (2018). Gender differences in repeated sprint ability. Isokinetics and Exercise Science, 26(1), 73–80. 10.3233/IES-180171191

[ref39] Toti, L., Bartalucci, A., Ferrucci, M., Fulceri, F., Lazzeri, G., Lenzi, P., Soldani, P., Gobbi, P., La Torre, A., & Gesi, M. (2013). High-intensity exercise training induces morphological and biochemical changes in skeletal muscles. Biology of Sport, 30(4), 301–309. 10.5604/20831862.1077557 24744502 PMC3944543

[ref40] Turner, A. N., & Stewart, P. F. (2013). Repeat sprint ability. Strength & Conditioning Journal, 35(1), 37–41. 10.1519/SSC.0b013e3182824ea4

[ref41] Ulupınar, S., Hazır, T., & Kin İşler, A. (2023). The contribution of energy systems in repeated-sprint protocols: The effect of distance, rest, and repetition. Research Quarterly for Exercise and Sport, 94(1), 173–179. 10.1080/02701367.2021.1950902 34781827

[ref42] Ulupınar, S., & Özbay, S. (2022). Energy pathway contributions during 60-second upper-body Wingate test in Greco-Roman wrestlers: intermittent versus single forms. Research in Sports Medicine, 30(3), 244–255. 10.1080/15438627.2021.1895784 33663306

[ref43] Ulupınar, S., Özbay, S., Gençoğlu, C., Franchini, E., Kishalı, N. F., & Ince, I. (2021). Effects of sprint distance and repetition number on energy system contributions in soccer players. Journal of Exercise Science & Fitness, 19(3), 182–188. 10.1016/j.jesf.2021.03.003 33889186 PMC8044429

[ref44] Wiecek, M., Szymura, J., Maciejczyk, M., Cempla, J., & Szygula, Z. (2016). Effect of sex and menstrual cycle in women on starting speed, anaerobic endurance and muscle power. Acta Physiologica Hungarica, 103(1), 127–132. 10.1556/036.103.2016.1.13 27030635

